# Modeling the role of microRNA-449a in the regulation of the G2/M cell cycle checkpoint in prostate LNCaP cells under ionizing radiation

**DOI:** 10.1371/journal.pone.0200768

**Published:** 2018-07-19

**Authors:** Shantanu Gupta, Daner A. Silveira, José Carlos M. Mombach

**Affiliations:** Department of Physics, Universidade Federal de Santa Maria, Santa Maria, Rio Grande do Sul, Brazil; University of South Alabama Mitchell Cancer Institute, UNITED STATES

## Abstract

Recent studies showed that induced microRNA-449a (miR-449a) enhances a G2/M cell cycle checkpoint arrest in prostate cancer (LNCaP) and lung adenocarcinoma cell lines. In the case of LNCaP cells, upregulated miR-449a directly downregulates c-Myc that is required to induce the cell cycle regulators Cdc25A and Cdc2/CyclinB whose inactivation blocks G2 to M phase transition. However, the molecular mechanisms involved are yet unclear, although in other prostate cancer cells the interactions among p53, miR-449a and Sirt-1 can affect the induction of the G2/M arrest. In order to clarify these molecular mechanisms, in this work we propose a boolean model of the G2/M checkpoint arrest regulation contemplating the influence of miR-449a. The model shows that the cell fate determination between two cellular phenotypes: G2/M-Arrest for DNA repair and G2/M-induced apoptosis is stochastic and influenced by miR-449a state of activation. The results were compared with experimental data available presenting agreement. We also found that several feedback loops are involved in this cell fate regulation and we indicate, through *in silico* gain or loss of function perturbations of genes, which of these feedback loops are more efficient to favor a specific phenotype.

## Introduction

**Fig 1:**
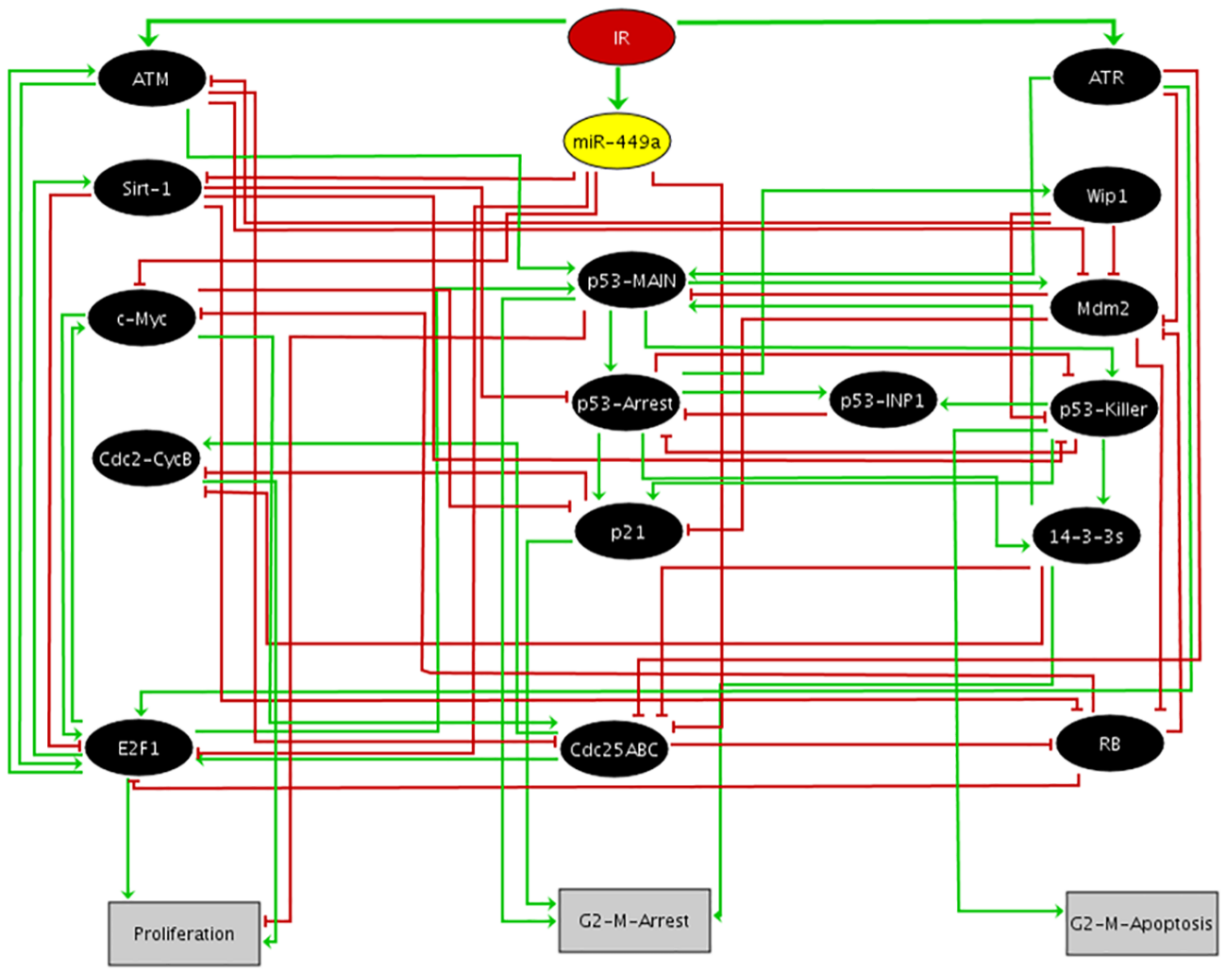
Regulatory network for G2/M checkpoint in response to IR. Elliptic nodes in black represent proteins and the yellow node is miR-449a. The input elliptic node in red denote IR (Ionizing Radiation), whereas rectangular light gray nodes represent model outputs (Proliferation, G2/M Arrest and Apoptosis). Green and red arcs denote positive and negative interactions, respectively.

It is well established that the p53 protein contributes to both G1/S and G2/M cell cycle checkpoint arrests in several cell types [1]. In LNCaP (prostate cancer) cell lines, a functional p53 contributes to increase cell survival under radiation response [2]. A recent paper by Mao et al. [3] suggests that miR-449a induces the G2/M checkpoint in LNCaP cells in response to ionizing radiation (IR). The work proposed a new induction mechanism for the G2/M checkpoint through downregulation of the Myc proto-oncogene protein (c-Myc) by miR-449a induction due to IR. Transfected mir-449a can also enhance the G2/M checkpoint activation and apoptosis in post-irradiated lung adenocarcinoma cells lines [4]. MicroRNAs are small, highly conserved, noncoding RNAs that negatively regulate protein expression and inhibit the translation of specific mRNAs. The miR-449 family of miRNAs (miR-449a, miR-449b and miR-449c) is located in the second intron of cell division cycle 20B (Cdc20B) gene located in chromosome 5. MiR-449a has decreased expression in several cancer cell lines [3, 5–8]. Under inhibited c-Myc, the cell cycle regulators: the cell division cycle 25 A (Cdc25A) and the complex formed by the cell division cycle protein 2 homolog and the Cyclin B complex (Cdc2/CyclinB) are not induced promoting a G2/M arrest. It was suggested that radiation can induce mir-449a directly [3], however we should expect that the DNA damage induced by radiation contributes to the induction of checkpoint arrest as well. In addition, in other prostate cancer cells the well known molecular interactions observed among p53, miR-449a and Sirtuin-1 (Sirt-1) might affect the regulation of G2/M arrest [9, 10]. Then, in order to bring more realism to the mechanisms involved in the induction of the G2/M checkpoint in LNCaP cells by mir-449a, in this work we propose a boolean model of this checkpoint regulation (Fig 1).

## Materials and methods

### The boolean formalism

Based on the published biochemical information, the approach starts with the definition of a regulatory graph, wherein each node represents a model component and each directed arc represents an (activatory or inhibitory) interaction between two components. The activities of all components are associated with boolean variables (taking only the values 0 or 1). The corresponding discretization reflects the ‘threshold effect’ of the regulatory interactions between components: a component is considered ‘active’ (1 or ON) as long as this activity level is sufficient to exert an effect, otherwise it is considered inactive (0 or OFF). Logical rules, based on the classical logical operators AND, OR, and NOT, define the evolution of the activity level of a component dependent on those of its regulators, see Table 1.

**Table 1:** Logical rules controlling the states of the nodes in Fig 1. The logical operators AND, OR and NOT are used to define the rules for each node in terms of the state of its regulators. There is a single boolean input, IR.

Nodes	Level	Rules	Biological interpretation
IR	1		Ionizing radiation present
ATM	1	IR *AND* (*NOT* Wip1 *OR* E2F1)	
ATR	1	IR	
miR-449a	1	IR	Activation by IR
Sirt-1	1	E2F1 *OR* *NOT* miR-449a	
p53-MAIN	1	(ATM *OR* ATR *OR* (E2F1 *AND* 14-3-3s)) *AND* *NOT* mdm2	p53 activation
Mdm2	1	(*NOT* Wip1 *OR* p53-MAIN *OR* RB) *AND* *NOT* ATM *AND* *NOT* ATR	
p53-Arrest	1	(p53-MAIN:1 *OR* *NOT* p53-INP1) *AND* *NOT* p53-Killer *AND* *NOT* Sirt-1	p53 phosphorylated at Ser-15 and Ser-20
p53-Killer	1	*NOT* p53-Arrest *AND* (*NOT* Sirt-1 *OR* *NOT* Wip1) *AND* p53-MAIN	p53 phosphorylated at Ser-15, Ser-20 and Ser-46
p53-INP1	1	p53-Arrest *OR* p53-Killer	Control of p53 accumulation
Wip1	1	p53-Arrest	
p21	1	(p53-Arrest *OR* p53-Killer) *AND* *NOT* c-Myc	
14-3-3s	1	p53-Arrest *OR* p53-Killer	
c-Myc	1	(E2F1 *OR* *NOT* RB) *AND* *NOT* miR-449a	
E2F1	1	(*NOT* RB *AND* ((ATM *AND* ATR *AND* *NOT* miR-449a) *OR* *NOT* Sirt-1)) *OR* c-Myc *OR* Cdc25ABC	
RB	1	*NOT* mdm2 *AND* *NOT* Cdc25ABC *AND* *NOT* Sirt-1	Dephosphorylated RB bound to E2F1
Cdc25ABC	1	*NOT* miR-449a *OR* c-Myc) *AND* *NOT* ATM *AND* *NOT* ATR *AND* *NOT* 14-3-3s	
Cdc2-CycB	1	Cdc25ABC *OR* (*NOT* p21 *AND* *NOT* 14-3-3s)	
Proliferation	1	*NOT* p53-MAIN *AND* (Cdc2-CycB *OR* E2F1)	p53-MAIN inhibition and activation of cycle regulators
G2/M-Arrest	1	p21 *OR* 14-3-3s	G2/M checkpoint arrest phenotype
G2/M-Apoptosis	1	p53-Killer	Apoptosis phenotype

There are two main types of regulatory circuits (also known as feedback loops): negative and positive circuits. Negative circuits can give rise to oscillations [11], whereas positive circuits are responsible for multistationarity [11]. The approach allows *in silico* gain (GoF) or loss of function (LoF) perturbations to test phenotype changes by forcing the value of a node to remain ON or OFF, respectively. Node values can be updated synchronously or asynchronously. In the synchronous case, all nodes are updated at the same time [12] and the evolution is completely deterministic. For the asynchronous case, random nodes are selected at each time and then updated [13], which can give rise to non deterministic behavior. Throughout this work, we have used only the asynchronous updating scheme.

The asynchronous update is defined through a regulatory network (*G*, *K*) containing a set of *n* regulatory components (representing molecules or biological processes), *G* = (*g*_1_, *g*_2_, … *g*_*n*_), where each *g*_*i*_ is boolean. *K* is a transition function defined as *K*(*g*) = (*K*_1_(*g*), … *K*_*n*_(*g*)), where each logical function, *K*_*i*_(*g*), defines the values of each *g*_*i*_ in terms of the influence of the edges that connect the *g*_*i*_. Mathematically, the asynchronous updating can be defined for all *i* ≠ *j* as:

$$\begin{array}{ccc} g_{i}(t+1) & = & g_{i}(t)+Sign\;[K_{i}(g_{i}(t))-g_{i}\left(t\right)]\\ \;g_{j}(t+1) & = & g_{j}(t). \end{array}$$ (1)

In this work we used the tool GINsim 2.9.5, which is a Java software suite, freely available for download from (http://compbio.igc.gulbenkian.pt/nmd/node/82) [14]. Using GINsim, we can compute the dynamical behavior of the model for any initial state. The state of each model component is iteratively updated, according to the logical formulae. The resulting dynamics is represented in terms of state transition graphs (STG). All probabilities of each phenotype presented in this study were computed using the Monte Carlo Algorithm provided by GINsim [14].

### G2/M checkpoint molecular mechanisms

The activation of the checkpoints in response to DNA damage induced by IR [15] is well established [16, 17]. Cycle arrest for repair or apoptosis can be triggered at G1/S and G2/M checkpoints. DNA double-strand breaks (DSB) and single-strand breaks (SSB) activate ATM and ATR, respectively. Phosphorylations downstream ATM and ATR pathways lead to the activation of p53 (reviewed by Gobbini [18]). In LNCaP cells line, miR-449a is required for the induction of the G2/M checkpoint which is our focus here, in fact LNCaP cells knockout for Mir-449a cannot arrest at G2/M [3]. We define our boolean model in terms of the molecular interactions involved in the activation of the G2/M checkpoint by DNA damage due to IR contemplating the interactions of miR-449a [19]. We also consider that IR can induce miR-449a directly as suggested by Mao et al. [3]. The model encompasses 21 nodes representing proteins or miRNAs and 61 direct interactions among them. Details about interactions and modeling techniques are presented in section Methods.

The model has a single input, IR (Fig 1). The logical rules controlling the nodes are presented in Table 1. Elliptic black nodes represent proteins and the yellow node represents miR-449a. Rectangular light gray nodes represent model outputs (Proliferation, G2/M Arrest and G2/M induced Apoptosis), green arrows denote activations and red hammerhead connectors denote inhibitions. In our model the representation of p53 was based on the work of Zhang et al. [15] that associates more than one node to p53 functions based on the different circuits that it participates. According to Zhang et al. model, p53 phosphorylated by ATM and ATR is represented by node p53-MAIN. While p53-Arrest and p53-killer nodes represent, respectively: p53 inducer of cell cycle arrest and p53 inducer of apoptosis. The Tumor protein p53 inducible nuclear protein 1 (p53-INP1) controls the conversion between p53-Arrest and p53-Killer. Additional details about these p53 nodes are given in what follows.

Activated or upregulated miR-449a suppresses proliferation in LNCaP cells and promotes apoptosis and cycle arrest by repressing c-Myc, E2F transcription factor 1 (E2F1), Cdc25ABC and Sirt-1 (Fig 1). It is important to point out that the reported activatory interaction of miR-449a by E2F1 for some cell lines, is not determinant for the state of the microRNA in LNCaP cells [3]. Then in the present model, as done in the work of Mao et al., it is ignored. Inhibition of Sirt-1 by miR-449a restores p53 (p53-MAIN) expression and promotes its acetylation [10]. E2F1 induces Sirt-1 expression and binds to E2F1 which binds to Retinoblastoma 1 protein (RB). E2F1 is directly inhibited by Sirt-1, forming a negative circuit (E2F1/Sirt-1) [20]. It was previously reported that functional p53-activated genes were only induced in LNCaP cells [2]. After DNA damage, p53 is phosphorylated by ATM and ATR. Activated p53 induces expression of E3 ubiquitin protein ligase homolog protein (Mdm2), which in turn, marks p53 for degradation, forming a negative-circuit (p53-Mdm2) [21]. In the model, based on the different circuits p53 participates, it is represented by different nodes: p53-MAIN associated with the interaction with Mdm2 which is required to activate p53-Arrest and p53-Killer. p53-Arrest representing p53 phosphorylated at Ser-15 and Ser-20, whereas p53-Killer represents p53 additionally phosphorylated at Ser-46 which leads to cell apoptosis [22]. p53-Arrest and p53-Killer are connected by a positive-circuit and the conversion between p53-Arrest and p53-Killer is regulated by Wip1 and p53-INP1 [15]. 14-3-3s also interacts with p53 forming a positive-circuit (14-3-3s/p53-MAIN) [23]. Binding to 14-3-3s protects p53 from Mdm2-mediated ubiquitylation, thereby stabilizing its levels and increasing its transcriptional activity. The cyclin-dependent kinase inhibitor 1A (p21), Protein Phosphatase 1D (Wip1), 14-3-3 Sigma (14-3-3s) and tumor protein p53 inducible nuclear protein 1 (p53-INP1) are activated by p53. Wip1 dephosphorylates ATM serine/threonine kinase (ATM). This action makes ATM activate p53-MAIN to the p53-Arrest form which activates transcription of Wip1, which in turn inactivates ATM by Wip1, another negative-circuit (Wip1/p53-MAIN/ATM) [24]. Cdc2/Cyclin B complex is directly inhibited by p21 and 14-3-3s. The mechanism by which p53 blocks cells at the G2/M checkpoint involves inhibition of Cdc2. p21 or 14-3-3s proteins sequester Cdc2 in the cytoplasm and directly block the activities of Cdc25 family, resulting in G2/M arrest [1].

References of each interaction and the GINsim version of the model can be found in the S1 and S2 Files, respectively. In addition, official names of the model elements can be found in the S1 Table.

## Results

### Attractors of the wild-type network

**Fig 2:**
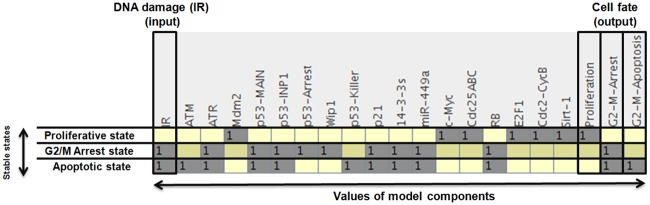
Stable states of the wild-type case. Left-most column represents IR levels and right-most columns represent outputs: Proliferation, G2/M-Arrest and G2/M-Apoptosis. Each line represent stable states corresponding to each IR level and phenotype, respectively. Empty spaces in each line represent zero values. Probabilities for wild type case states: 50% for Proliferation (without IR), 34% for G2/M-Apoptosis and 16% for G2/M-Arrest.

The model presents 3 states for the wild-type case dynamics which are associated to different phenotypes (Fig 2). The first state (IR = 0) is a proliferative stable state, due to activation only of the cell cycle promoters E2F1, Cdc2-CycB, c-Myc and Cdc25ABC. This state is controlled by the positive circuit c-Myc/E2F1 in the model [25], see Table 2. The second and third states (IR = 1) correspond to a bistable state involving two p53-responsive cellular phenotypes: G2/M-Arrest and G2/M-Apoptosis, that are defined by p53-Arrest and p53-Killer activations, respectively. The positive circuit encompassing these two nodes controls the bistability. As we show below, we find that miR-449a determines the functionality of this circuit and others such as p53/14-3-3s and RB/Mdm2, which are both positive, and the negative circuits: ATM/p53-MAIN/Wip1, p53-MAIN/Mdm2 and E2F1/Sirt-1. Then, if miR-449a is inactive, these circuits are not functional.

**Table 2:** Functional circuits in the model and experimental observations. Cases for which no experimental data were found are indicated by question marks.

Circuits	References
**Positive**	
RB/Mdm2	[27]
E2F1/ATM	[28]
E2F1/c-Myc	[25]
p53-MAIN/14-3-3s	[23]
p53-Killer/p53-Arrest	[15]
**Negative**	
p53-MAIN/Mdm2	[21]
p53-INP1/p53-Arrest	[15]
ATM/p53-MAIN/Wip1	[24]
Sirt-1/E2F1	[20]
E2F1/Cdc25ABC/ATM	?

To estimate the size of the basin of attraction of the states of the wild type case in Fig 2, we sampled the space of states using a Monte Carlo algorithm (Exact Exit Probabilities) over 1000 runs [26] to calculate the probability of each phenotype (see section Methods). We obtained that the probability of the proliferative state is 50%, of G2/M-Apoptosis is 34% and of G2/M-Arrest is 16%.

### Model validation

The model validation was conducted through simulations of node perturbations to investigate the correspondence between stable states of the model and experimental observations. In this way, we constructed Table 3 to show the agreement between our results and experimental observations [3]. For more details about model validation results see S3 File.

**Table 3:** The model agrees with all experimental results from Mao et al. [3]. E1 represents GoF and KO represents LoF of the corresponding gene.

Stimulus/Perturbations	Response/phenotype
MiR-449 in response to IR	Upregulation
miR-449a KO	Proliferation
miR-449a E1	Inhibits proliferation/Induce cell cycle arrest and apoptosis
MiR-449 and Myc in response to IR	Negative correlation
Myc KO	G2/M arrest, Apoptosis

### Role of functional circuits and phenotype probabilities

GINsim identified only 10 functional circuits in the model, see the Table 2. Seven of them have their functionality controlled by miR-449a as mentioned above. We decided to investigate how these circuits affect the bistable behavior regulated by miR-449a to determine if it was possible to control the probabilities of the two non proliferative phenotypes (G2/M-Arrest or G2/M-Apoptosis) in the model. To do that we used perturbations, the results are shown in Table 4. We considered only the perturbations where miR-449a is activated since we want to unravel its role in the regulatory process. We calculated the probabilities of each phenotype using the Monte Carlo algorithm with 1000 runs as previously mentioned. We also determined that p53-MAIN activation and the positive circuit p53-Arrest/p53-killer are required to produce the bistability. In this way, we considered only perturbations of circuits where p53-MAIN, p53-Arrest and p53-killer are involved. In all results we found a higher probability to obtain apoptosis (although other circuit perturbations can produce the opposite result (see S4 File). As we can see in Table 4, the circuit ATM/p53-MAIN/Wip1 has the stronger effect in terms of controlling the probabilities of each phenotype.

**Table 4:** Perturbations of the circuits whose functionality is controlled by miR-449a that affect the probabilities of the bistable dynamics in the model. Probabilities were calculated using a Monte Carlo algorithm with 1000 runs in GINsim. E1 represents GoF and KO represents LoF of the corresponding element of the circuit.

Circuits		miR-449a	
Negative	Perturbations	E1	
		Phenotype	Probability
ATM/p53-MAIN/Wip1	KO/E1/KO	G2/M or Apoptosis	25%, 75%
	E1/E1/KO	G2/M or Apoptosis	25%, 75%
	KO/E1/E1	G2/M or Apoptosis	37%, 63%
	E1/E1/E1	G2/M or Apoptosis	37%, 63%
p53-MAIN/Mdm2	E1/KO	G2/M or Apoptosis	21%, 79%
E2F1/Sirt-1	KO/KO	G2/M or Apoptosis	31%, 69%
	E1/KO	G2/M or Apoptosis	36%, 64%
**Positive**			
RB/Mdm2	E1/KO	G2/M or Apoptosis	29%, 71%
p53-MAIN/14-3-3s	E1/KO	G2/M or Apoptosis	22%, 78%
	E1/E1	G2/M or Apoptosis	25%, 75%

We found that our model can also present oscillatory dynamics for some specific perturbations not presented in Table 4 [21, 24]. Interestingly, we also observed that perturbations of the E2F1/ATM and E2F1/Cdc25ABC/ATM circuits, whose functionality is not controlled by miR-449a, can have a higher effect on the probability of obtaining G2/M-Arrest. According to our model, these circuits have their functionality affected by p53-MAIN and RB, respectively, See S4 File.

## Discussion and conclusion

In this study, we proposed a logical model of G2/M-arrest regulation in LNCaP cells focusing on the role of miR-449a. Our model describes the induction of G2/M arrest and apoptosis in terms of two IR-responsive mechanisms according to the literature [3, 9, 10]: DNA damage and miR-449a. The p53 pathway is induced by DNA damage and miR-449a can be induced directly by IR as suggested by Mao et al. [3]. These two pathways must crosstalk through Sirt-1 since this was already observed in other prostate cells [9, 10]. Sirt-1 functionality is controlled by miR-449a and it can indirectly affect p53 dynamics. Indeed, Kheir et al. [29], showed that miR-449a can induce p53 by repression of Sirt-1 leading to cell apoptosis.

In the absence of IR, the wild type case (Fig 2) predicts only a proliferative phenotype which is supported by the work of Mao et al. [3]. However, when DNA damage due to IR is present, miR-449a is induced activating the p53 pathway and one of the possible outcomes, G2/M Arrest (p53-Arrest) or Apoptosis (p53-Killer). The role of miR-449a and p53 in the induction of G2/M arrest and apoptosis in LNCaP cells in our model is characterized in Table 4. These results show that miR-449a controls several circuits and that p53 is essential to induce bistability between these phenotypes through the p53-Arrest/p53-Killer circuit. This result agrees with Zhang et al. [15] work that show a two-phase dynamics of p53 in response to IR. Moreover, the model predicts that the system favors an apoptotic phenotype for the bistability case in response to IR according to the probabilities. This type of bistable control of phenotypes that we propose here was already used to explain cell fate determination between apoptosis or senescence at the G1/S checkpoint observed in some non-Hodgkin lymphoma cells [30].

In summary, the model agrees with experimental results and predicts that miR-449a activates several circuits that produce oscillations and bistability. The only disagreement between our model and Mao’s et al. [3] is that our model favors apoptosis rather than G2/M arrest in response to IR.

Thus, through exploration of the mechanisms involved in induction of apoptosis and G2/M arrest phenotypes in LNCaP cells, our work provides a tool to help improve cancer therapy.

## Supporting information

S1 File Molecular interactions. Bibliographical references of the molecular interactions in the model.
(PDF)

S2 File GINsim code. The GINsim 2.9.5 code of the model.
(ZIP)

S3 File Model validation. Model comparison with experimental results.
(PDF)

S4 File Additional circuit perturbations. *In silico* perturbations of circuits that make G2/M-arrest more probable than apoptosis.
(PDF)

S1 Table Molecule official names. Official names of the model elements.
(PDF)
