# Supplementary material for: Modeling the role of microRNA-449a in the regulation of the G2/M cell cycle checkpoint in prostate LNCaP cells under ionizing radiation
Source: PLoS One. 2018 Jul 19;13(7):e0200768. doi: 10.1371/journal.pone.0200768 (PMC6053189; doi:10.1371/journal.pone.0200768)
Supplement: S1 File — Bibliographical references of the molecular interactions in the model. (PDF) [file pone.0200768.s001.pdf]

Modeling the role of microRNA-449a in the regulation of the G2/M cell cycle checkpoint in prostate LNCaP cells under ionizing radiation
S1 file

Shantanu Gupta[1,+], Daner A. Silveira[1,+] and José C. M. Mombach[1,+,*]

[1]Departamento de Física, Universidade Federal de Santa Maria, Santa Maria, RS, 97105-900, Brazil

[*]Corresponding author (e-mail: jcmombach@ufsm.br)
[+]These authors contributed equally to this work

Bibliographical references of the interactions among the molecules of the model

IR → miR-449a [1, 2]
IR → ATR [3]
IR → ATM [3]
p21 → G2/M-Arrest [4]
p53-Main → p53-Arrest [5]
p53-Main → p53-Killer [6]
p53-Main → Mdm2 [7]
p53-Main → G2/M-Arrest [8]
14-3-3s → G2/M-Arrest [9]
Cdc25ABC → E2F1 [10]
Myc → Cdc25ABC [11]
Myc → E2F1 [12]
E2F1 → Myc [12]
E2F1 → Sirt-1 [13]
E2F1 → p53-Main [14]
E2F1 → Proliferation [15]
ATM → E2F1 [16]
ATM → p53 [17]
ATR → E2F1 [16]
ATR → p53 [17]
E2F1 → ATM [18]
Cdc25ABC → Cdc2-CycB [19]
Cdc2-CycB → Proliferation [20]
p53-Killer → 14-3-3 [21]
p53-Killer → p21 [22, 8]
p53-Killer → G2/M-Apoptosis [23]
p53-Arrest → 14-3-3 [21]
p53-Arrest → p21 [22, 8]
p53-Arrest → Wip1 [24]
p53-Arrest → p53INP1 [25]
p53INP1 ⊣ p53-Arrest [25]
p21 ⊣ Cdc2-CycB [26]
p53-Main ⊣ Proliferation [27]
p53-Arrest ⊣ p53-killer [28]
p53-Killer ⊣ p53-Arrest [28]
MDM2 ⊣ RB [29]
MDM2 ⊣ P53-Main [7]
MDM2 ⊣ [30]
Wip1 ⊣ p53-Killer [31]
Wip1 ⊣ mdm2 [31]
Wip1 ⊣ ATM [32]
ATM ⊣ Cdc25ABC [33]
ATM ⊣ Mdm2 [34]

ATR ⊣ Mdm2 [35]
ATR ⊣ Cdc25ABC [33]
Sirt-1 ⊣ p53-Arrest [36]
Sirt-1 ⊣ p53-Killer [36]
Sirt-1 ⊣ E2F1 [13]
Sirt-1 ⊣ RB [37]
miR-449a ⊣ Myc [1, 2]
c-Myc ⊣ p21 [38]
miR-449a ⊣ Cdc25ABC [39]
Cdc25ABC ⊣ Rb [40]
miR-449a ⊣ Sirt-1 [41]
sirt-1 ⊣ P53 [42]
14-3-3 ⊣ Cdc2-CycB [43]
Rb ⊣ E2F1 [44]
Rb ⊣ c-Myc [45]
Rb ⊣ mdm2 [46]
miR-449a ⊣ E2F1 [39]

References

[1] Aihong Mao, Qiuyue Zhao, Xin Zhou, Chao Sun, Jing Si, Rong Zhou, Lu Gan, and Hong Zhang. MicroRNA-449a enhances radiosensitivity by downregulation of c-Myc in prostate cancer cells. *Scientific reports*, 6:27346, 2016.

[2] Aihong Mao, Yang Liu, Yali Wang, Qiuyue Zhao, Xin Zhou, Chao Sun, Cuixia Di, Jing Si, Lu Gan, and Hong Zhang. MiR-449a enhances radiosensitivity through modulating pRb/E2F1 in prostate cancer cells. *Tumor Biology*, 37(4):4831–4840, 2016.

[3] Jeremy S Myers and David Cortez. Rapid activation of ATR by ionizing radiation requires ATM and Mre11. *Journal of Biological Chemistry*, 281(14):9346–9350, 2006.

[4] Alexander B Niculescu, Xinbin Chen, Monique Smeets, Ludger Hengst, Carol Prives, and Steven I Reed. Effects of p21Cip1/Waf1 at both the G1/S and the G2/M cell cycle transitions: pRb is a critical determinant in blocking DNA replication and in preventing endoreduplication. *Molecular and cellular biology*, 18 (1):629–643, 1998.

[5] Natalia S Pellegata, Ronald J Antoniono, J Leslie Redpath, and Eric J Stanbridge. DNA damage and p53-mediated cell cycle arrest: a reevaluation. *Proceedings of the National Academy of Sciences*, 93(26):15209–15214, 1996.

[6] E Christine Pietsch, Stephen M Sykes, Steven B McMahon, and Maureen E Murphy. The p53 family and programmed cell death. *Oncogene*, 27(50):6507–6521, 2008.

[7] Ute M Moll and Oleksi Petrenko. The MDM2-p53 interaction. *Molecular cancer research*, 1(14):1001–1008, 2003.

[8] William R Taylor and George R Stark. Regulation of the G2/M transition by p53. *Oncogene*, 20(15):1803, 2001.

[9] Heiko Hermeking, Christoph Lengauer, Kornelia Polyak, Tong-Chuan He, Lin Zhang, Sam Thiagalingam, Kenneth W Kinzler, and Bert Vogelstein. 14-3-3σ is a p53-regulated inhibitor of G2/M progression. *Molecular cell*, 1(1):3–11, 1997.

[10] Tao Shen and Shile Huang. The role of Cdc25A in the regulation of cell proliferation and apoptosis. *Anti-Cancer Agents in Medicinal Chemistry (Formerly Current Medicinal Chemistry-Anti-Cancer Agents)*, 12(6):631–639, 2012.

[11] S Aref, M Fouda, E El-Dosoky, A Menessy, M Mabed, M Saleeb, and K Zalata. C-Myc oncogene and Cdc25A cell activating phosphatase expression in non-hodgkin's lymphoma. *Hematology*, 8(3):183–190, 2003.

[12] JY Leung, GL Ehmann, PH Giangrande, and JR Nevins. A role for Myc in facilitating transcription activation by E2F1. *Oncogene*, 27(30):4172–4179, 2008.

[13] Chuangui Wang, Lihong Chen, Xinghua Hou, Zhenyu Li, Neha Kabra, Yihong Ma, Shino Nemoto, Toren Finkel, Wei Gu, W Douglas Cress, et al. Interactions between E2F1 and SirT1 regulate apoptotic response to DNA damage. *Nature cell biology*, 8(9):1025–1031, 2006.

[14] Jung-Kuang Hsieh, Damian Yap, Daniel J O'Connor, Valentina Fogal, Lynn Fallis, Florence Chan, Shan Zhong, and Xin Lu. Novel function of the cyclin a binding site of E2F in regulating p53-induced apoptosis in response to DNA damage. *Molecular and cellular biology*, 22(1):78–93, 2002.

[15] Sanghoon Lee, Jung Nyeo Chun, Su-Hwa Kim, Insuk So, and Ju-Hong Jeon. Icilin inhibits E2F1-mediated cell cycle regulatory programs in prostate cancer. *Biochemical and biophysical research communications*, 441(4):1005–1010, 2013.

[16] Weei-Chin Lin, Fang-Tsyr Lin, and Joseph R Nevins. Selective induction of E2F1 in response to DNA damage, mediated by ATM-dependent phosphorylation. *Genes & development*, 15(14):1833–1844, 2001.

[17] DNA damage sensing by the ATM] and ATR kinases, author=Maréchal, Alexandre and Zou, Lee, journal=Cold Spring Harbor perspectives in biology, volume=5, number=9, pages=a012716, year=2013, publisher=Cold Spring Harbor Lab.

[18] Eli Berkovich and Doron Ginsberg. ATM is a target for positive regulation by E2F-1. *Oncogene*, 22(2):161–167, 2003.

[19] Oleg Timofeev, Onur Cizmecioglu, Florian Settele, Tore Kempf, and Ingrid Hoffmann. Cdc25 phosphatases are required for timely assembly of CDK1-cyclin B at the G2/M transition. *Journal of Biological Chemistry*, 285(22):16978–16990, 2010.

[20] Hisanori Kawamoto, Hirotaka Koizumi, and Toshiyuki Uchikoshi. Expression of the G2-M checkpoint regulators cyclin B1 and cdc2 in nonmalignant and malignant human breast lesions: immunocytochemical and quantitative image analyses. *The American journal of pathology*, 150(1):15, 1997.

[21] Heng-Yin Yang, Yu-Ye Wen, Chih-Hsin Chen, Guillermina Lozano, and Mong-Hong Lee. 14-3-3σ positively regulates p53 and suppresses tumor growth. *Molecular and cellular biology*, 23(20):7096–7107, 2003.

[22] Guangan He, Zahid H Siddik, Zaifeng Huang, Ruoning Wang, John Koomen, Ryuji Kobayashi, Abdul R Khokhar, and Jian Kuang. Induction of p21 by p53 following DNA damage inhibits both Cdk4 and Cdk2 activities. *Oncogene*, 24 (18):2929–2943, 2005.

[23] Susan Haupt, Michael Berger, Zehavit Goldberg, and Ygal Haupt. Apoptosis-the p53 network. *Journal of cell science*, 116(20):4077–4085, 2003.

[24] Mutsuhiro Takekawa, Masaaki Adachi, Atsuko Nakahata, Ichiro Nakayama, Fumio Itoh, Hiroyuki Tsukuda, Yoichi Taya, and Kohzoh Imai. p53-inducible Wip1 phosphatase mediates a negative feedback regulation of p38 MAPK-p53 signaling in response to UV radiation. *The EMBO journal*, 19(23):6517–6526, 2000.

[25] Shu Okamura, Hirofumi Arakawa, Tomoaki Tanaka, Hiroshi Nakanishi, Ching Ching Ng, Yoichi Taya, Morito Monden, and Yusuke Nakamura. p53DINP1, a p53-inducible gene, regulates p53-dependent apoptosis. *Molecular cell*, 8(1):85–94, 2001.

[26] Ludger Hengst, Ulrich Göpfert, Hilal A Lashuel, and Steven I Reed. Complete inhibition of Cdk/cyclin by one molecule of p21Cip1. *Genes & development*, 12 (24):3882–3888, 1998.

[27] Joongkyu Park, Yohan Oh, Lang Yoo, Min-Su Jung, Woo-Joo Song, Sang-Hun Lee, Hyemyung Seo, and Kwang Chul Chung. Dyrk1A phosphorylates p53 and inhibits proliferation of embryonic neuronal cells. *Journal of Biological Chemistry*, 285(41):31895–31906, 2010.

[28] Steven E Artandi and Laura D Attardi. Pathways connecting telomeres and p53 in senescence, apoptosis, and cancer. *Biochemical and biophysical research communications*, 331(3):881–890, 2005.

[29] Zhi-Xlong Xiao, Jiandong Chen, Arnold J Levine, Nazanlne Modjtahedi, Jun Xing, William R Sellers, and David M Livingston. Interaction between the retinoblastoma protein and the oncoprotein MDM2. *Nature*, 375(6533):694, 1995.

[30] Luciana E Giono and James J Manfredi. Mdm2 is required for inhibition of Cdk2 activity by p21, thereby contributing to p53-dependent cell cycle arrest. *Molecular and cellular biology*, 27(11):4166–4178, 2007.

[31] Xiongbin Lu, Ou Ma, Thuy-Ai Nguyen, Stephen N Jones, Moshe Oren, and Lawrence A Donehower. The Wip1 phosphatase acts as a gatekeeper in the p53-Mdm2 autoregulatory loop. *Cancer cell*, 12(4):342–354, 2007.

[32] Sathyavageeswaran Shreeram, Oleg N Demidov, Weng Kee Hee, Hiroshi Yamaguchi, Nobuyuki Onishi, Calvina Kek, Oleg N Timofeev, Crissy Dudgeon, Albert J Fornace, Carl W Anderson, et al. Wip1 phosphatase modulates ATM-dependent signaling pathways. *Molecular cell*, 23(5):757–764, 2006.

[33] Maria Thanasoula, Jose Miguel Escandell, Natsuko Suwaki, and Madalena Tarsounas. ATM/ATR checkpoint activation downregulates CDC25C to prevent mitotic entry with uncapped telomeres. *The EMBO journal*, 31(16):3398–3410, 2012.

[34] Ruth Maya, Moshe Balass, Seong-Tae Kim, Dganit Shkedy, Juan-Fernando Martinez Leal, Ohad Shifman, Miri Moas, Thomas Buschmann, Ze'ev Ronai, Yosef Shiloh, et al. ATM-dependent phosphorylation of Mdm2 on serine 395: role in p53 activation by DNA damage. *Genes & development*, 15(9):1067–1077, 2001.

[35] Tomomi Shinozaki, Ayumi Nota, Yoichi Taya, and Koji Okamoto. Functional role of Mdm2 phosphorylation by ATR in attenuation of p53 nuclear export. *Oncogene*, 22(55):8870–8880, 2003.

[36] Jingjie Yi and Jianyuan Luo. SIRT1 and p53, effect on cancer, senescence and beyond. *Biochimica et Biophysica Acta (BBA)-Proteins and Proteomics*, 1804(8): 1684–1689, 2010.

[37] Beata Jablonska, Marcin Gierdalski, Li-Jin Chew, Teresa Hawley, Mackenzie Catron, Arturo Lichauco, Juan Cabrera-Luque, Tracy Yuen, David Rowitch, and Vittorio Gallo. Sirt1 regulates glial progenitor proliferation and regeneration in white matter after neonatal brain injury. *Nature communications*, 7:13866, 2016.

[38] Andrei L Gartel, Xin Ye, Eugene Goufman, Pavel Shianov, Nissim Hay, Feridoon Najmabadi, and Angela L Tyner. Myc represses the p21 (WAF1/CIP1) promoter and interacts with Sp1/Sp3. *Proceedings of the National Academy of Sciences*, 98(8):4510–4515, 2001.

[39] Xiaojing Yang, Min Feng, Xia Jiang, Zhenlong Wu, Zhimei Li, Meiyee Aau, and Qiang Yu. MiR-449a and miR-449b are direct transcriptional targets of E2F1 and negatively regulate pRb-E2F1 activity through a feedback loop by targeting CDK6 and CDC25A. *Genes & development*, 23(20):2388–2393, 2009.

[40] Maddalena Donzelli and Giulio F Draetta. Regulating mammalian checkpoints through Cdc25 inactivation. *EMBO reports*, 4(7):671–677, 2003.

[41] Parameet Kumar, Shashwat Sharad, Gyorgy Petrovics, Ahmed Mohamed, Albert Dobi, Taduru L Sreenath, Shiv Srivastava, and Roopa Biswas. Loss of miR-449a in ERG-associated prostate cancer promotes the invasive phenotype by inducing SIRT1. *Oncotarget*, 7(16):22791, 2016.

[42] Ling Li, Lisheng Wang, Liang Li, Zhiqiang Wang, Yinwei Ho, Tinisha McDonald, Tessa L Holyoake, WenYong Chen, and Ravi Bhatia. Activation of p53 by SIRT1 inhibition enhances elimination of CML leukemia stem cells in combination with imatinib. *Cancer cell*, 21(2):266–281, 2012.

[43] Paulette Mhawech. 14-3-3 proteins—an update. *Cell research*, 15(4):228–236, 2005.

[44] SW Hiebert, SP Chellappan, JM Horowitz, and JR Nevins. The interaction of rb with E2F coincides with an inhibition of the transcriptional activity of E2F. *Genes & development*, 6(2):177–185, 1992.

[45] Roderick L Beijersbergen, E Marielle Hijmans, Liang Zhu, and Rene Bernards. Interaction of c-Myc with the pRb-related protein p107 results in inhibition of c-Myc-mediated transactivation. *The EMBO journal*, 13(17):4080, 1994.

[46] Damian BS Yap, Jung-Kuang Hsieh, Florence SG Chan, and Xin Lu. mdm2: a bridge over the two tumour suppressors, p53 and Rb. *Oncogene*, 18(53):7681, 1999.
