# Supplementary material for: Modeling the role of microRNA-449a in the regulation of the G2/M cell cycle checkpoint in prostate LNCaP cells under ionizing radiation
Source: PLoS One. 2018 Jul 19;13(7):e0200768. doi: 10.1371/journal.pone.0200768 (PMC6053189; doi:10.1371/journal.pone.0200768)
Supplement: S3 File — Model comparison with experimental results. (PDF) [file pone.0200768.s003.pdf]

Modeling the role of microRNA-449a in the regulation of the G2/M cell cycle checkpoint in prostate LNCaP cells under ionizing radiation
S3 file

Shantanu Gupta[1,+], Daner A. Silveira[1,+] and José C. M. Mombach[1,+,*]

[1]Departmento de Física, Universidade Federal de Santa Maria, Santa Maria, RS, 97105-900, Brazil

[*]Corresponding author (e-mail: jcmombach@ufsm.br)
[+]These authors contributed equally to this work

List of stable states of the model in section Model validation

IR	ATM	ATR	Mdm2	p53-MAIN	p53-INP1	p53-Arrest	Wip1	p53-Killer	p21	14-3-3s	miR-449a	c-Myc	Cdc25ABC	RB	E2F1	Cdc2-CycB	Sirt-1	Proliferation	G2-M-Arrest	G2-M-Apoptosis
			1									1	1		1	1	1	1		
1		1		1	1	1	1		1	1	1			1					1	
1	1	1		1	1			1	1	1	1			1					1	1

Figure 1: Wild type case

IR	ATM	ATR	Mdm2	p53-MAIN	p53-INP1	p53-Arrest	Wip1	p53-Killer	p21	14-3-3s	miR-449a	c-Myc	Cdc25ABC	RB	E2F1	Cdc2-CycB	Sirt-1	Proliferation	G2-M-Arrest	G2-M-Apoptosis
			1									1	1		1	1	1	1		
1	1	1		1	1			1		1		1			1		1		1	1

Figure 2: Knockout (KO) of miR-449a

IR	ATM	ATR	Mdm2	p53-MAIN	p53-INP1	p53-Arrest	Wip1	p53-Killer	p21	14-3-3s	miR-449a	c-Myc	Cdc25ABC	RB	E2F1	Cdc2-CycB	Sirt-1	Proliferation	G2-M-Arrest	G2-M-Apoptosis
1		1		1	1	1	1		1	1	1			1					1	
1	1	1		1	1			1	1	1	1			1					1	1

Figure 3: Ectopic expression (E1) of miR-449a, Inhibits proliferation and induce G2/M-Arrest and apoptosis

IR	ATM	ATR	Mdm2	p53-MAIN	p53-INP1	p53-Arrest	Wip1	p53-Killer	p21	14-3-3s	miR-449a	c-Myc	Cdc25ABC	RB	E2F1	Cdc2-CycB	Sirt-1	Proliferation	G2-M-Arrest	G2-M-Apoptosis
			1									1	1		1	1	1	1		
1		1		1	1	1	1		1	1	1			1					1	
1	1	1		1	1			1	1	1	1			1					1	1

Figure 4: Negative correlation between c-Myc and miR-449a in response to IR

IR	ATM	ATR	Mdm2	p53-MAIN	p53-INP1	p53-Arrest	Wip1	p53-Killer	p21	14-3-3s	miR-449a	c-Myc	Cdc25ABC	RB	E2F1	Cdc2-CycB	Sirt-1	Proliferation	G2-M-Arrest	G2-M-Apoptosis
			1										1		1	1	1	1		
1		1		1	1	1	1		1	1	1			1					1	
1	1	1		1	1			1	1	1	1			1					1	1

Figure 5: Knockout (KO) of c-Myc induce G2/M-Arrest and Apoptosis
