# Supplementary material for: Modeling the role of microRNA-449a in the regulation of the G2/M cell cycle checkpoint in prostate LNCaP cells under ionizing radiation
Source: PLoS One. 2018 Jul 19;13(7):e0200768. doi: 10.1371/journal.pone.0200768 (PMC6053189; doi:10.1371/journal.pone.0200768)
Supplement: S4 File — In silico perturbations of circuits that make G2/M-arrest more probable than apoptosis. (PDF) [file pone.0200768.s004.pdf]

Modeling the role of microRNA-449a in the regulation of the G2/M cell cycle checkpoint in prostate LNCaP cells under ionizing radiation

S4 file

Shantanu Gupta[1,+], Daner A. Silveira[1,+] and José C. M. Mombach[1,+,*]

[1]Departamento de Física, Universidade Federal de Santa Maria, Santa Maria, RS, 97105-900, Brazil

[*]Corresponding author (e-mail: jcmombach@ufsm.br)
[+]These authors contributed equally to this work

Other positive and negative circuits with functionality affected by p53-MAIN and RB (and not regulated by miR-449a). These circuit perturbations make G2/M-arrest more probable than apoptosis. Probabilities were calculated using the Monte Carlo algorithm (see section Methods).

Table 1: Perturbations of the positive circuit regulated by p53-MAIN. E1 represents GoF and KO represents LoF of the corresponding element of the circuit.

**Circuits**		**p53-MAIN**	
**Positive**	**Perturbations**	**E1**	
		Phenotype	Probability
E2F1/ATM	KO/KO	G2/M or Apoptosis	71%, 29%
	KO/E1	G2/M or Apoptosis	72%, 28%

Table 2: Perturbations of the negative circuit regulated by RB. E1 represents GoF and KO represents LoF of the corresponding element of the circuit.

**Circuit**		**RB**	
**Negative**	**Perturbations**	**E1**	
		Phenotype	Probability
E2F1/Cdc25ABC/ATM	KO/KO/KO	G2/M or Apoptosis	71%, 29%
	KO/KO/E1	G2/M or Apoptosis	70%, 30%
	KO/E1/KO	G2/M or Apoptosis	69%, 31%
	KO/E1/E1	G2/M or Apoptosis	70%, 30%
