# Supplementary material for: Modeling the role of microRNA-449a in the regulation of the G2/M cell cycle checkpoint in prostate LNCaP cells under ionizing radiation
Source: PLoS One. 2018 Jul 19;13(7):e0200768. doi: 10.1371/journal.pone.0200768 (PMC6053189; doi:10.1371/journal.pone.0200768)
Supplement: S1 Table — Official names of the model elements. (PDF) [file pone.0200768.s005.pdf]

Modeling the role of microRNA-449a in the regulation of the G2/M cell cycle checkpoint in prostate LNCaP cells under ionizing radiation
S1 Table

Shantanu Gupta[1,+], Daner A. Silveira[1,+] and José C. M. Mombach[1,+,*]

[1]Departmento de Física, Universidade Federal de Santa Maria, Santa Maria, RS, 97105-900, Brazil

[*]Corresponding author (e-mail: jcmombach@ufsm.br)
[+]These authors contributed equally to this work

Model elements

Table 1: Model elements / corresponding molecules or cytotoxic agent

Nodes	Description
IR	Ionizing radiation
ATM	ATM serine/threonine kinase
ATR	ATR serine/threonine kinase
miR-449a	MicroRNA 449a
Sirt-1	Sirtuin 1
p53-MAIN	Tumor supressor p53 protein
Mdm2	E3 ubiquitin protein ligase homolog protein
p53-Arrest	Tumor supressor p53 protein (Ser-15 and Ser-20)
p53-Killer	Tumor supressor p53 protein (Ser-46)
p53-INP1	Tumor protein p53 inducible nuclear protein 1
Wip1	Protein Phosphatase 1D
p21	cyclin-dependent kinase inhibitor 1A
14-3-3s	14-3-3 Sigma
c-Myc	Myc proto-oncogene protein
E2F1	E2F transcripition factor 1
RB	Retinoblastoma 1 protein
Cdc25ABC	Cell division cycle 25 family (A/B/C)
Cdc2-CycB	Cell division cycle protein 2 homolog/ Cyclin B complex
